# Bigfin reef squid demonstrate capacity for conditional discrimination and projected future carbon dioxide levels have no effect on learning capabilities

**DOI:** 10.7717/peerj.9865

**Published:** 2020-09-29

**Authors:** Blake L. Spady, Sue-Ann Watson

**Affiliations:** 1 Australian Research Council Centre of Excellence for Coral Reef Studies, James Cook University, Townsville, QLD, Australia; 2 College of Science and Engineering, James Cook University, Townsville, QLD, Australia; 3 Biodiversity and Geosciences Program, Museum of Tropical Queensland, Queensland Museum, Townsville, QLD, Australia

**Keywords:** Cephalopod, Climate change, Conditional discrimination, Learning, Ocean acidification, Squid

## Abstract

Anthropogenic carbon dioxide (CO_2_) emissions are being absorbed by the oceans, a process known as ocean acidification, and risks adversely affecting a variety of behaviours in a range of marine species, including inhibited learning in some fishes. However, the effects of elevated CO_2_ on learning in advanced invertebrates such as cephalopods are unknown. Any impacts to the learning abilities of cephalopods could have far-reaching consequences for their populations and the communities they inhabit. Cephalopods have some of the most advanced cognitive abilities among invertebrates and are one of the few invertebrate taxa in which conditional discrimination has been demonstrated, though the trait has not been demonstrated in any species of squid. Here, we tested for the first time the capacity for conditional discrimination in a squid species (*Sepioteuthis lessoniana*). Furthermore, we investigated the effects of projected future CO_2_ levels (1,084 µatm) on conditional discrimination and learning more generally. A three-task experiment within a two-choice arena was used to test learning and conditional discrimination. Learning was measured by improvements in task completion in repeated trials over time and the number of trials required to pass each task. Squid exhibited significant learning capabilities, with an increase in correct choices over successive trials and a decrease in the number of trials needed to complete the successive tasks. Six of the 12 squid tested successfully passed all three tasks indicating a capacity for conditional discrimination in the species. Elevated CO_2_ had no effect on learning or on the capacity for conditional discrimination in squid. This study highlights the remarkable cognitive abilities of *S. lessoniana*, demonstrated by their capacity for conditional discrimination, and suggests that ocean acidification will not compromise learning abilities. However, other behavioural traits in the species have been shown to be altered at comparable elevated CO_2_ conditions. It is not clear why some ecologically important behaviours are altered by elevated CO_2_ whereas others are unaffected. Future research should focus on the physiological mechanism responsible for altered behaviours in squid at elevated CO_2_.

## Introduction

The coleoid cephalopods (squids, cuttlefishes, and octopuses), when compared to other invertebrates, show remarkably advanced learning and memory abilities (Dickel, Boal & Budelmann, 1999). Their complex nervous systems and highly diverse behaviours have been compared to those of lower vertebrates (i.e. fishes and amphibians), despite considerable evolutionary distance between these taxonomic groups (Boycott & Young, 1950; Boycott, 1961; Hanlon & Messenger, 2018). The morphology, physiology, ecology, and behaviours of coleoid cephalopods (henceforth referred to as cephalopods) were shaped by a coevolutionary arms race with modern teleost fishes (Mather & Kuba, 2013), resulting in many abilities and traits that both groups share (Packard, 1972; O’Dor & Webber, 1986). Among these are advanced eyes, large brains, and capacity for visual conditional discrimination. While conditional discrimination has been demonstrated in cuttlefish and octopuses (Hvorecny et al., 2007; Mather & Kuba, 2013), whether squid possess this trait has not been investigated.

Conditional discrimination is the ability to control discernment between different options through a sensitivity to context, which has significant benefits to individual performance (Mackay, 1991). For example, if reinforcement is delivered when an individual presses a square button and not a triangular button, discrimination is required for success. However, if reinforcement is contingent upon pushing the square button only after hearing the auditory stimulus ‘square’, conditional discrimination is necessary. In this example the auditory stimulus is the conditional stimulus, and the button shape is the discriminative stimulus. This allows for two initially unrelated stimuli to be associated with a single event, causing an emergent relationship to develop between those stimuli (Zentall, 1998). Conditional discrimination is considered ‘complex learning’ (Thomas, 1980, 1996) and has only been demonstrated in a small number of invertebrate species. Aside from in cuttlefishes (*Sepia pharaonis* and *Sepia officinalis*) and a species of octopus (*Octopus bimaculoides*), conditional discrimination has only been demonstrated in two other invertebrate classes, the sea hare, *Aplysia californica* (Colwill, Absher & Roberts, 1988), and the bees, *Apis mellifera* and *Bombus impatiens* (Couvillon & Bitterman, 1988; Brown et al., 1998; Mirwan & Kevan, 2015). The high level learning required for conditional discrimination undoubtedly contributes to the continued ecological success of the species, benefiting hunting, predator avoidance, social interactions, and navigation (Hanlon & Messenger, 2018). Thus, despite the paucity of studies, it is likely that conditional discrimination is common among a range of highly social or predatory animal taxa, such as squid.

Squid occupy an important mesopredator role in marine food webs as they both prey upon a wide range of marine species and are predated on by an even more diverse variety of species (Hanlon & Messenger, 2018). In mesopredators, the use of discrimination is important for both hunting and predator avoidance (MacDougall & Dawkins, 1998). In a predator capacity, all cephalopods detect prey predominately by sight and hunt with a wide array of techniques that vary depending on prey type (Boycott, 1954; Ross, 1971; Curio, 1976; Ross & Von Boletzky, 1979). Squid can use subtle visual cues to discern weaker or slower individuals within schooling fishes, ensuring greater capture success (Neill & Cullen, 1974). In a prey capacity, learning to identify predators can influence survival as predator recognition is not always innate knowledge (Brodie, Formanowicz & Brodie, 1991; Mitchell et al., 2011). Predator recognition also often involves fine tuning the efficiency of anti-predator responses by learning which predators are a substantive threat and which are not (Ferrari & Chivers, 2011).

Discrimination also supports spatial orientation, especially in benthic associated species such as octopuses and cuttlefishes (Hvorecny et al., 2007). Advanced conditional discrimination and long-term memory has been demonstrated in a number of cephalopod species (e.g. octopuses and cuttlefishes) using maze experiments (Schiller, 1949; Wells, 1964, 1967; Walker, Longo & Bitterman, 1970; Boal et al., 2000; Karson, Boal & Hanlon, 2003; Alves et al., 2006, 2007; Hvorecny et al., 2007). Compared to the mazes in laboratory experiments, conditional discrimination supporting spatial orientation in the wild often differentiates between much more specific and subtle cues within an unstable environment (Forsythe & Hanlon, 1997). The importance and level of complexity of spatial learning among species depends on their lifestyle and habitat type, but is expected to be widespread among animals (Capaldi, Robinson & Fahrbach, 1999).

Recently, projected future levels of carbon dioxide (CO_2_) from increased anthropogenic activity have been shown to alter learning in some marine species (Ferrari et al., 2012; Chivers et al., 2014). Atmospheric concentrations of CO_2_ have increased by more than 40% since the Industrial Revolution and the partial pressure of CO_2_ (*p*CO_2_) in the oceans rises at the same rate, a process known as ocean acidification (Dlugokencky & Tans, 2019). If CO_2_ emissions continue at the current rate, atmospheric CO_2_ could exceed 900 ppm by the end of this century (RCP8.5) (Collins et al., 2013). Furthermore, seasonal fluctuations in seawater *p*CO_2_ are projected to be amplified with increasing CO_2_ concentrations due to the increased Revelle (buffer) factor of acidified seawater (McNeil & Sasse, 2016). CO_2_ concentrations matching these worst case scenarios for the end of this century elicit a range of altered behavioural responses in a variety of marine taxa (Clements & Hunt, 2015; Nagelkerken & Munday, 2016), including inhibited learning in fishes (Ferrari et al., 2012; Chivers et al., 2014).

Two species of tropical squid are among the animals that exhibit altered behaviour at elevated CO_2_. The two-toned pygmy squid, *Idiosepius pygmaeus*, responds to elevated CO_2_ with altered escape responses (Spady et al., 2014). Furthermore, *I. pygmaeus* and bigfin reef squid (*Sepioteuthis lessoniana*) respond to elevated CO_2_ with altered predatory behaviours, such as increased latency to attack prey, and increased activity (Spady et al., 2014; Spady, Munday & Watson, 2018). Both in their predatory and anti-predator responses, these species displayed altered body pattern choice at elevated CO_2_ (Spady et al., 2014; Spady, Munday & Watson, 2018). However, whether elevated CO_2_ also affects learning in these species is unknown.

While the effects of elevated CO_2_ on learning have not been investigated in any invertebrate species, research into the effects of elevated CO_2_ on learning in fishes suggests a potential for similar adverse effects in other marine taxa. The tropical damselfish, *Pomacentrus amboinensis*, failed to learn to appropriately respond to a common predator fish after 4 days exposure to elevated CO_2_ (~850 µatm) (Ferrari et al., 2012). In the temperate fish, *Gasterosteus aculeatus*, learning and memory is also affected by elevated CO_2_ (~1,000 µatm), with reduced arena escape times over successive trials (Jutfelt et al., 2013). The effects of elevated CO_2_ on learning in reef fishes have been linked to impaired function of GABA_A_ receptors, a major inhibitory neurotransmitter receptor (Chivers et al., 2014). In squid, GABA_A_-like receptors and other inhibitory mechanisms are also important for learning processes (Conti et al., 2013). For example GABA_A_ is likely to be involved in the experience-dependent learning of prey capture and escape responses, which is dependent on the inhibitory control of the giant synapse output (Preuss & Gilly, 2000). While the relationship between CO_2_ induced altered behaviour and GABA_A_ has not been investigated in squid, the effects of elevated CO_2_ on the predator escape responses in the gastropod mollusc, the humpbacked conch *Gibberulus gibberulus gibbosus*, has been linked to interference with GABA_A_-like receptors (Watson et al., 2014), which suggests that learning could also be affected by elevated CO_2_ in molluscs.

In this study, we investigate the capacity for conditional discrimination in the bigfin reef squid and the effect of elevated CO_2_ on their learning. Although conditional discrimination or associative learning has not been previously demonstrated in a species from the order of *S. lessoniana* (Teuthida), a wide range of cephalopod species appear to share advanced learning capabilities (Boal, 1996; Hvorecny et al., 2007; Karson, Boal & Hanlon, 2003; Zepeda, Veline & Crook, 2017). Furthermore, while conditional discrimination has not been demonstrated in many invertebrate species, it does occur in the relatively simple-“brained” sea hare, *A. californica* (Colwill, Absher & Roberts, 1988). Therefore, we expect that conditional discrimination is likely more widespread throughout the animal kingdom than is currently recognised, and we expect the trait is present in squid as well.

*Sepioteuthis lessoniana* was chosen due to their close association with coral reef habitats (Norman, 2003), indicating a high prioritisation of spatial orientation when compared to more pelagic squid species. The species is highly active and has one of the largest global distributions of any inshore squid, found throughout temperate and tropical waters from Japan to northern Australia and New Zealand and from Hawaii to the east African coast, north into the Red Sea and south to southern Mozambique and Madagascar (Jereb & Roper, 2005). The genus *Sepioteuthis* has been noted for their complex learned social and mating behaviours (Moynihan & Rodaniche, 1982; Hanlon & Messenger, 2018; Sugimoto & Ikeda, 2012). Furthermore, the previously demonstrated effects of elevated CO_2_ on activity levels and predatory behaviours (Spady, Munday & Watson, 2018) make *S. lessoniana* an ideal subject for this study.

In the present study, squid were held at control (502 µatm) or elevated CO_2_ (1,084 µatm) seawater treatments and subjected to repeated trials in a two-choice arena experiment with three separate tasks to complete, closely following the methods of Karson, Boal & Hanlon (2003) and Hvorecny et al. (2007) used for demonstrating conditional discrimination in cuttlefishes. Squid were trained using visual cues in repeated motivation-based trial and error tasks with the goal of using the designated exit. Learning was measured as improvements in trial completion over time as well as the number of trials needed to pass each task. By completing all three tasks, squid will have displayed a capacity for conditional discrimination. If learning or the potential conditional discrimination capabilities of *S. lessoniana* are altered by elevated CO_2_, this could have far-reaching effects by adversely affecting the prey capture, predator avoidance, navigation, migration, or social interactions of an ecologically important species.

## Materials and Methods

### Animal collection and care

Twelve *S. lessoniana* (85–192 mm mantle length) were collected by dip-net in May and June 2017 from the Townsville breakwater, QLD, Australia. Collection permits from the Queensland Department of Agriculture, Fisheries and Forestry were obtained for animal collection (Permit Number: 170251). Animals were captured at night with a dip-net (2.5 cm mesh diameter) and transported immediately to the research aquarium facility at James Cook University, Townsville. Squid were kept individually in round tanks (47Ø × 51H cm; white colour) filled to 67-L with a continuous flow of 100% oxygenated natural seawater within a recirculating system. Squid remained at control conditions for 14 days before being relocated to different tanks of the same size and volume, which received a continuous flow of either control or elevated CO_2_ treatment water. All tanks received natural light, but were protected from direct sunlight by an opaque plastic roof. Squid were fed a single live fish or prawn (various species) three times daily. Individuals were maintained at either control (*n* = 6) or elevated CO_2_ (*n* = 6) for 40 days before learning trials were initiated. This treatment duration represents approximately 20% of the species’ average 208-day lifespan in the wild (Walsh et al., 2002). Colorimetric testing kits were used weekly to ensure suitable levels of ammonium (<0.50 ppm), nitrite (<0.50 ppm), and nitrate (<10 ppm) were maintained. Mantle length of individuals were measured to the nearest millimetre at the conclusion of the experiment using a fabric measuring tape.

### CO_2_ treatment systems

Experiments were conducted using two 8,000 L recirculating seawater systems at James Cook University’s research aquarium in Townsville, QLD, Australia. Each system provided UV sterilisation, dual bag filters (50 µm), and bio-ball filtration. Experimental treatments were a current-day ambient control (502 µatm CO_2_) and an elevated CO_2_ treatment (1,084 µatm CO_2_) consistent with upper end-of-century projections for RCP8.5 (Collins et al., 2013). The target goal for the control treatment was 410 µatm CO_2_. As pH measurements were taken directly from tanks containing animals, we expect that the difficulty in reaching this target may have been due to the increased rate of respiration of algae during the darker winter months. Each experimental treatment supplied seawater directly to six of the twelve 67 L squid holding tanks, which were divided evenly among three separate 1,000 L tanks as a water bath. The desired pH level in the elevated CO_2_ treatment was achieved with a custom-built pH control system, which dosed CO_2_ into 3,000 L sumps. An inline ISFET pH sensor (Tophit CPS471D; Endress+Hauser, Reinach, Switzerland) measured pH continuously and communicated with a computerised controller (OMNI C40 BEMS; Innotech, Brisbane, QLD, Australia) and regulated the CO_2_ dosing with a solenoid valve. Inline pH sensors were calibrated monthly with buffers pH 4.01 and pH 7.00. Daily measurements of pH on the NBS scale (pH_NBS_) were recorded (Seven2Go Pro; Mettler Toledo, Greifensee, Switzerland) from a random squid tank from each system and dosing set points were subsequently adjusted in order to maintain the target *p*CO_2_ in each treatment. The temperature was measured daily from a random squid tank in each system (C26; Comark, Norfolk, UK). Each squid tank received equilibrated seawater from their system at a rate of 1.5 L min^−1^.

Weekly water samples were analysed by spectrophotometry (UVmini-1240; Shimadzu Suzhou Instruments Co. Ltd., Kyoto, Japan) using m-cresol purple as an indicator dye (Dickson & Millero, 1987; Dickson, Sabine & Christian, 2007) in order to determine pH on the total scale (pH_T_). Gran Titration (888 Titrando; Metrohm AG, Herisau, Switzerland) was utilised weekly to estimate total alkalinity. All calibrations of the titrator remained within one percent of the known value of certified reference material from Dr. A.G. Dickson (Scripps Institution of Oceanography, batch #135). Weekly salinity measurements were taken with a conductivity sensor (HQ15d; Hach, Loveland, CO, USA). CO2SYS (Pierrot, Lewis & Wallace, 2006) was used to calculate carbonate chemistry parameters (Table 1) using the constants K1, K2 from Mehrbach et al. (1973) refit by Dickson & Millero (1987) and Dickson, Sabine & Christian (2007) for KHSO_4_.

**Table 1:** Seawater carbonate chemistry. The temperature, salinity, pH_(T)_, total alkalinity, and *p*CO_2_ for each treatment. Values are means ± SD.

Treatment	Temperature (C°)	Salinity	pH_(T)_	Total alkalinity (µmol kg^−1^ SW)	*p*CO_2_ (µatm)
Control	26.8 (±0.8)	36.1 (±0.7)	7.98 (±0.03)	2,464 (±65)	502 (±26)
Elevated-CO_2_	26.5 (±0.5)	35.8 (±0.5)	7.68 (±0.06)	2,345 (±123)	1,084 (±155)

### Testing arena

**Figure 1:**
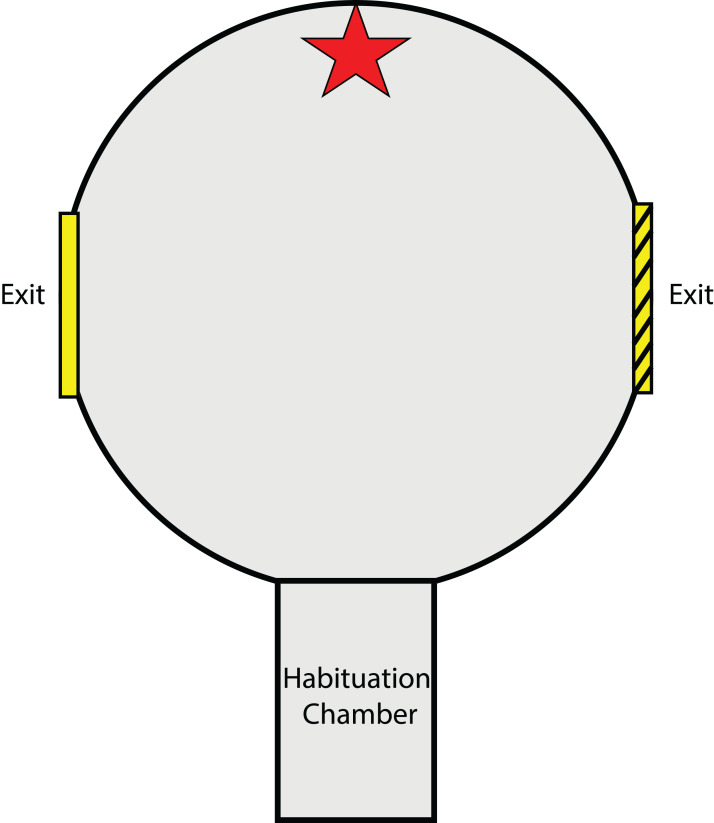
Testing arena as viewed from above. Red star indicates location of visual object cue (either a red brick or plastic aquarium plant). Exits were placed on the left and right side of the arena and were fitted with a visual exit frame for reference (either solid yellow or yellow with black stripes). The habituation chamber included a rising gate that allowed squid to enter the arena after the habituation period.

The testing arena and methods for the experiment described in this section closely resemble those of Karson, Boal & Hanlon (2003) and Hvorecny et al. (2007) designed for cuttlefish. A circular testing arena (56Ø × 40H cm) was constructed from dark grey PVC (Fig. 1). As with the experiments of Karson, Boal & Hanlon (2003) and Hvorecny et al. (2007), this is a two-choice exit arena, providing visual cues (object cues and exit frame) to teach individuals which exit is open and which is closed. A rectangular habituation chamber (27 × 15 cm) with a liftable gate, located at one end of the arena (equidistant and 90° from both exit points), was used to contain the animal before each trial. Upon entering the centre of the arena from the habituation chamber, the animal could view two circular exits (20 cm Ø and raised 10 cm from the base of the arena) located directly to the left and to the right. Visual object cues were placed directly opposite the habituation chamber gate except during preference testing trials. The arena and surrounding tank were filled with seawater to 29 cm deep (~500 L), leaving the top one cm of the exit above the water surface. This was done to avoid abrasions to the animals when passing through the exit as many individuals preferred to swim at the surface during trials.

Attached by Velcro around each exit was a square laminated exit frame (34 × 34 cm) (Karson, Boal & Hanlon, 2003; Hvorecny et al., 2007). One exit frame was solid yellow in colour while the other was yellow with black stripes. These two exit frames were alternated between the left and right exit for different individuals. A piece of clear Plexiglass™ that did not distort polarisation (Shashar, Rutledge & Cronin, 1996) was used to block the desired exit from the outside of the arena and was held in place by a brick at the base of the Plexiglass™, below the exit. Another brick was also placed in the same position outside of the unobstructed exit so as not to give any distinguishing characteristics to the blocked exit. Blocked exits were used to train squid to use a particular exit given a specific visual cue. In the experiments of Karson, Boal & Hanlon (2003) and Hvorecny et al. (2007), an arena with a transparent bottom was suspended above the tank floor in an attempt to encourage cuttlefish, which preferred staying on the substrate, to exit the arena to reach the floor. In this experiment however, preliminary tests of squid revealed an adverse reaction to the dark enclosure of the arena and a preference for areas of the tank with no obstructions above them. This instead was used as motivation for squid to escape the enclosed arena by navigating out one of the two exits. Therefore, a plywood board served as a lid to cover the top of the circular testing arena, while areas surrounding the arena were open and not covered. Trials were viewed via the display of a video camera (HC-V160; Panasonic, Osaka, Japan) which filmed downwards through a hole in the plywood board.

### Learning trials

#### General procedures

At the beginning of each trial, squid were placed in the habituation chamber for 1 min, after which the gate to the arena was opened. Preliminary trials within the arena determined that a 1-min habituation time was enough for squid to resume normal swimming behaviour and postures (no defensive arm postures) when entering the arena. Squid were given 30 s to leave the habituation chamber and enter the arena after the gate was opened. At the end of this period of time, if they did not enter the arena, they would be gently guided out of the habituation chamber with a dip-net. All squid had to be guided out of the habituation chamber at least once during preference testing. After exiting the habituation chamber, the gate was closed behind them. Once squid had entered the arena, to encourage the individual to exit the arena, a dip-net was lowered directly in front of the habituation chamber gate 20 s after the animal entered the arena (Hvorecny et al., 2007). After an additional 10 s, the net was lifted up and down at a set rate (full up/down cycle every 2 s), using a stop watch, until the squid had exited the arena (Hvorecny et al., 2007). Each animal was given 3 min to exit the arena once they had entered; if they did not exit the arena within this time, they were marked as having failed the trial and were guided out of an unobstructed exit with a dip-net.

For each of the three tasks described below, individuals were given a maximum of 60 trials per task to successfully pass and proceed to the next task. Criteria for passing tasks are described in the following sections. If a squid could not pass a task within the 60 trials, they would be marked as having failed that task and would not continue on to the subsequent task. Individuals performed up to seven trials per day with an inter-trial interval of 45 min. Animals were tested in their treatment water, which was continuously supplied to the main tank outside of the testing arena.

#### Preference testing

During preference testing, both exits were unobstructed and there was no visual object cue. Placement of the two distinct exit frames was chosen at random for each squid (whether left exit or right exit) and remained on the same exit for all trials and tasks for that individual. Preference testing was complete when an individual had: (a) exited the maze in under 3 min in six out of seven consecutive trials, and (b) used both the left and right exit at least once. The use of both exits was necessary in order to ensure the animal was aware that both exits lead to the same area. Preference was defined as the side (left or right) through which the squid passed through most frequently in these six trials (Karson, Boal & Hanlon, 2003; Hvorecny et al., 2007). If both exits were used equally after six consecutive trials, another trial would be performed to determine preference. A preference was considered strong if individuals chose the same exit in five or more of the consecutive trials.

#### Tasks 1 & 2: Learning

In Task 1 (testing against preference), individuals were tested against their preferred exit, meaning the exit they preferred in preference testing was blocked off with the Plexiglass™ barrier. During this task, an object cue (either a red brick or a green plastic aquarium plant) was introduced by placing it directly opposite the entrance from the habituation chamber. For Task 1, half of the animals from each treatment were presented the red brick as a visual cue, while the other half were presented the green plastic aquarium plant. This arrangement provided squid with two visual indications to learn which exit was unobstructed: the object cue and the exit frames.

To successfully pass Task 1, as well as subsequent tasks, individuals were required to exit the arena, in less than 3 min, in six out of seven consecutive trials without first attempting to pass through the blocked exit by touching the Plexiglass™ barrier with their mantle. If Task 1 was completed, the opposite object cue replaced the initial one within the arena, and the opposite exit was blocked (Task 2—testing with preference). Aside from the difference in visual object cue and which exit was obstructed, Task 2 followed the same procedures as in Task 1. Changes in correct exit choice and time to exit the arena over successive trials within tasks, as well as number of trials performed before passing each task were used to compare the learning performances among individuals and between experimental treatments.

#### Task 3: Conditional discrimination

Finally, if Task 2 was completed, squid were tested in trials in which the presented object cue (and the corresponding blocked exit as per Tasks 1 and 2) was semi-randomised (Task 3) by flipping a coin, but ensuring that the same object cue was not presented more than four times consecutively. In Task 3, squid had to determine which exit to use based on learned knowledge from the previous two tasks and distinguish which exit and frame would not be blocked by taking note of the object cue presented. By successfully choosing the correct exit in six out of seven consecutive trials before the failure cut-off of 60 total trials, individuals were deemed to have demonstrated conditional discrimination by controlling their exit choice by a sensitivity to the context of presented visual cues. In the experiment, the object cue (aquarium plant or red brick) is the conditional stimulus, and the exit frame (solid yellow or yellow with black stripes) is the discriminative stimulus. This study followed animal ethics guidelines from the James Cook University Animal Ethics Committee (JCU Animal Ethics Number: A2189).

### Statistical analyses

Statistical analyses were performed using R statistical software (R Development Core Team, 2020). To analyse any potential effect of CO_2_ treatment on individual ability to pass each task, a generalised linear model (GLM) with a binomial distribution was used for each task. Here, preference strength (number of exits through preferred side during preference testing) and visual cue were used as explanatory fixed factors. To determine the effect of elevated CO_2_ on the number of trials needed to pass each task, a GLM with a negative binomial distribution was used for each task. The difference in mean time for each individual to exit the arena in each task was compared between CO_2_ treatments with a GLM with a Gamma distribution.

Repeated measures generalised linear mixed-effects models (GLMM) with negative binomial distributions were used to analyse the time to exit the arena over successive trials within each task between CO_2_ treatments. Repeated measures GLMMs with binomial distributions were used for trends in the successful exit choice over successive trials between CO_2_ treatments. These repeated measures analyses included mantle length (indicative of animal age) and preference strength as explanatory variables and squid ID and trial number as a random effect. Repeated measures GLMMs were also used to compare the differences in mean time to exit the arena (Gaussian with log link), number of trials needed to meet passing criteria (Poisson distribution), and percent of correct exit choices (Gaussian with logit transformation) among tasks, as well as to examine effects of CO_2_ treatment on these relationships. These repeated measures analyses included preference strength as an explanatory variable and squid ID as the random effect.

Differences in preference strength between CO_2_ treatments were analysed with a linear regression model (LME). Among all individuals, the effect of mean time taken to exit the arena in Task 1 and the ability to successfully reach Task 3, as well as the effect of number of trials needed to pass Task 1 on the ability to reach and complete Task 3, were analysed with a GLM with a binomial distribution. A binomial probability distribution was used to determine the probability of choosing the correct exit six times in a series of seven trials by chance. A Cohen’s d power analysis was performed on the probability of finding an effect of CO_2_ treatment on the ability for individuals to successfully demonstrate conditional discrimination. Residual analysis indicated that data met the assumptions of normality and homogeneity of variance given the specified distributions chosen.

## Results

### All individuals

All 12 squid completed preference testing, taking between 9 and 30 trials. Four squid from the control treatment exhibited a strong preference towards one exit (5–6 uses of the same exit) and two squid from the elevated CO_2_ treatment exhibited a strong preference towards one exit. There was no effect of elevated CO_2_ on the preference strength of squid (GLM, χ^2^ = 1.000, *p* = 0.317). All 12 animals met the passing criteria for Task 1 (testing against preference), taking between 7 and 38 trials. Seven individuals met the passing criteria for Task 2 (testing with preference) (between 7 and 26 trials). CO_2_ treatment had no significant effect on their success in Task 2; four individuals were from the control treatment and three were from the elevated CO_2_ treatment (GLM, χ^2^ = 0.707, *p* = 0.401). Of the seven squid to reach Task 3 (testing for conditional discrimination), one of the four individuals from the control treatment failed to meet the passing criteria for Task 3, while all three squid from the elevated CO_2_ treatment successfully met the passing criteria for this task; CO_2_ treatment had no significant effect (GLM, χ^2^ = 1.243, *p* = 0.265). Six of the twelve squid tested successfully completed all three tasks, indicating that *S. lessoniana* has a capacity for conditional discrimination. Of squid that demonstrated conditional discrimination, all individuals met the criteria for passing Task 3 in between 7 and 11 trials. Three of these squid were from the control treatment and three were from the elevated CO_2_ treatment. Elevated CO_2_ had no effect on the ability of squid to demonstrate conditional discrimination (GLM, χ^2^ = 0.024, *p* = 0.876).

**Figure 2:**
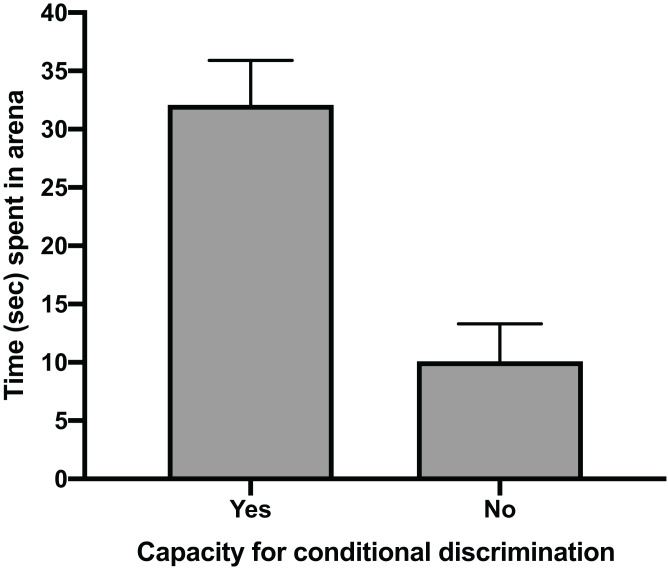
Time spent in arena. The mean time in seconds (±SE) spent in the testing arena during Task 1 (testing against preference) trials for individuals that passed Task 3 (testing for conditional discrimination) by demonstrating a capacity for conditional discrimination compared to those that did not reach or pass Task 3.

**Figure 3:**
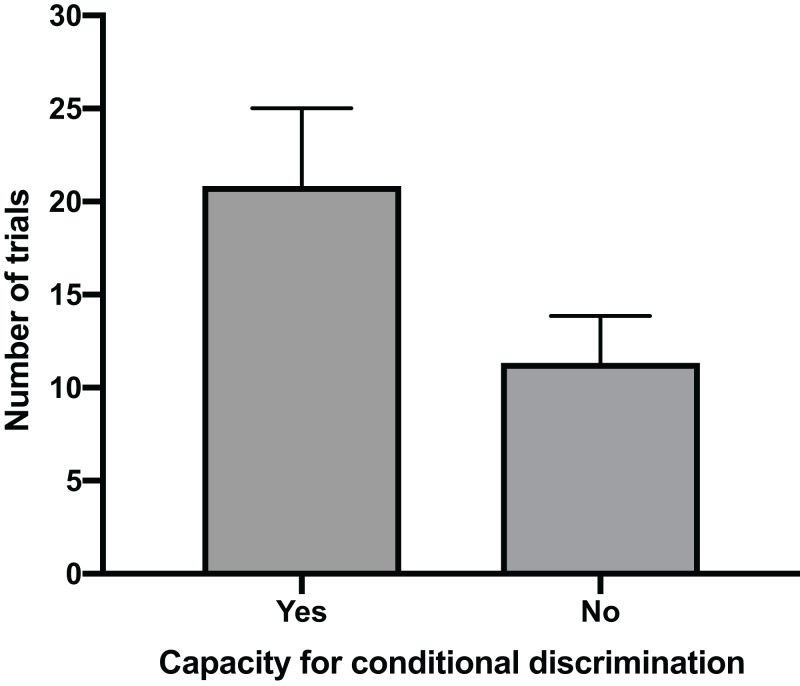
Number of trials to pass Task 1. The mean number of trials (±SE) required to successfully complete Task 1 for individuals that passed Task 3 by demonstrating a capacity for conditional discrimination compared to those that did not reach or pass Task 3.

The mean time for individuals to exit the arena in Task 1 (testing against preference) had a clear positive relationship with their ability to reach Task 3 (testing for conditional discrimination) (GLM, χ^2^ = 6.705, *p* = 0.010). Individuals that successfully reached Task 3 had a mean exit time in Task 1 trials of 32.1 s (±5.2 SE), whereas individuals that did not reach Task 3 spent only 10.1 s (±4.9 SE) in the arena during Task 1 trials (Fig. 2). There was also a positive relationship between the number of trials performed to pass Task 1 and the ability to pass Task 3 (GLM, χ^2^ = 4.053, *p* = 0.044). The mean number of trials needed to pass Task 1 in individuals that passed Task 3 (20.8 ± 4.2 SE) was nearly double that of individuals that failed to pass Task 3 (11.3 ± 2.5 SE) (Fig. 3). All subsequent results focus only on the six individuals that successfully passed Task 3.

### Individuals that demonstrated conditional discrimination

**Figure 4:**
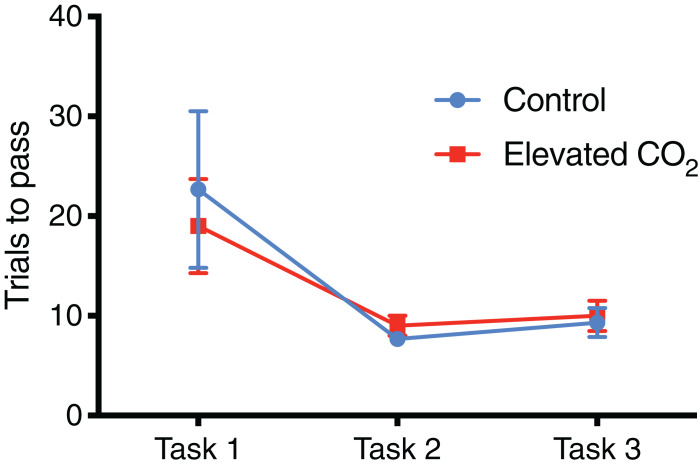
Number of trials for each task. The mean number of trials (±SE) performed before successfully meeting the passing criteria for each task in control and elevated CO_2_ squid for those individuals that successfully passed all three tasks.

**Figure 5:**
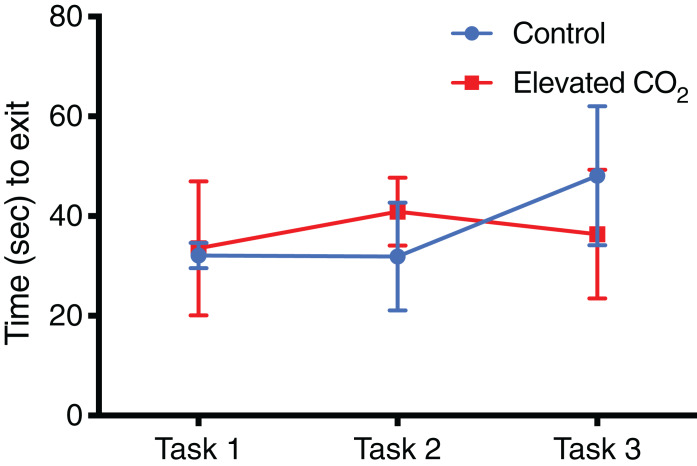
Time spent in arena for each task. The mean exit time (±SE) in successful trials for each task in control and elevated CO_2_ squid for those individuals that successfully passed all three tasks.

For individuals that successfully passed Task 3 and demonstrated conditional discrimination, the number of trials needed to successfully complete each task did not differ between CO_2_ treatments (Fig. 4): Task 1 (GLM, χ^2^ = 0.262, *p* = 0.609), Task 2 (GLM, χ^2^ = 0.320, *p* = 0.571), Task 3 (GLM, χ^2^ = 0.069, *p* = 0.793). There was also no difference between CO_2_ treatments in the mean time to exit the arena within each task (Fig. 5): Task 1 (GLM, χ^2^ = 0.012, *p* = 0.914), Task 2 (GLM, χ^2^ = 0.433, *p* = 0.511), Task 3 (GLM, χ^2^ = 0.371, *p* = 0.542). Improvements in exit times and correct exit choices of squid over successive trials within each task were observed in some tasks, but did not significantly differ between CO_2_ treatments. In Task 1, the time for squid to exit the arena did not significantly decrease over successive trials (LME, χ^2^ = 0.426, *p* = 0.514), and there was no effect of CO_2_ treatment on this relationship (LME, χ^2^ = 0.909, *p* = 0.340). There was an improvement in correct exit choice for squid over successive trials in Task 1 (GLMM, χ^2^ = 5.806, *p* = 0.016), but this relationship was not influenced by CO_2_ treatment (GLMM, χ^2^ = 0.073, *p* = 0.787). There was no significant improvement in exit time in Task 2 over successive trials for squid (LME, χ^2^ = 0.431, *p* = 0.511) and no effect of CO_2_ treatment on this relationship (LME, χ^2^ = 2.499, *p* = 0.114). Similar to Task 1, there was also a significant improvement in Task 2 in the correct exit chosen over successive trials (GLMM, χ^2^ = 6.617, *p* = 0.010); CO_2_ treatment had no effect on this relationship (GLMM, χ^2^ = 1.960, *p* = 0.162). In Task 3, the time to exit the arena over successive trials for all squid did not improve (LME, χ^2^ = 1.499, *p* = 0.221) and there was no effect of CO_2_ treatment (LME, χ^2^ = 2.005, *p* = 0.157). While there was a trend in Task 3 of increasing correct choices over successive trials, this was not significant (GLMM, χ^2^ = 3.117, *p* = 0.078) and there was again no effect of elevated CO_2_ (GLMM, χ^2^ = 0.018, *p* = 0.892).

**Figure 6:**
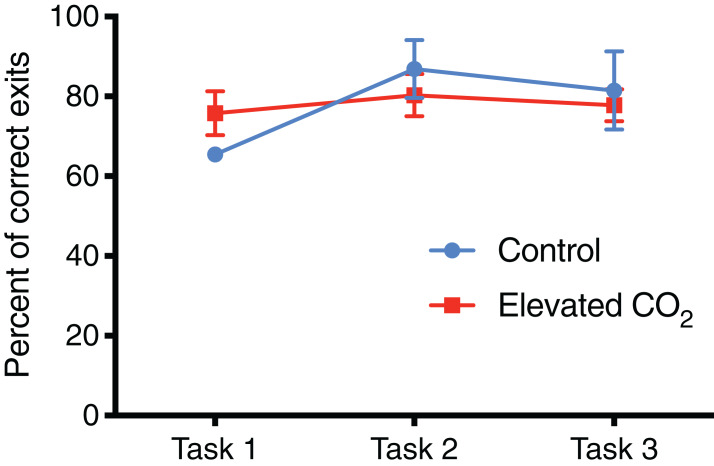
Percentage of correct exit choices. The mean percentage of trials (±SE) in which squid chose the correct exit within each task for control and elevated CO_2_ squid for those individuals that passed all three tasks. The error for control animals at Task 1 is very small and cannot be visualised at this scale.

In individuals that successfully passed Task 3, there was a significant decrease in the number of trials needed to complete each task from Task 1 (mean of 21 trials) to Tasks 2 and 3 (mean of 8 and 10 trials, respectively) (GLMM, χ^2^ = 40.842, *p* < 0.001) and elevated CO_2_ had no effect on this relationship (GLMM, χ^2^ = 1.249, *p* = 0.535). The mean time to exit the arena did not change significantly among Tasks 1, 2, and 3 (LME, χ^2^ = 4.446, *p* = 0.108), and there was also no effect of elevated CO_2_ (LME, χ^2^ = 3.981, *p* = 0.137). The percentage of trials within each task in which individuals chose the correct exit increased from Task 1 (71% correct) to Tasks 2 and 3 (84% and 80% correct, respectively) but this was not significant (GLMM, χ^2^ = 2.919, *p* = 0.232), and there was no difference between CO_2_ treatments (GLMM, χ^2^ = 2.103, *p* = 0.350) (Fig. 6). The probability of meeting the passing criteria for each task by chance alone (six correct exits out of seven trials) is 0.055. A power analysis revealed a power of 0.22 on the effect of CO_2_ treatment on the ability to demonstrate conditional discrimination.

## Discussion

Of the 12 bigfin reef squid (*S. lessoniana*) tested, six successfully completed the test for conditional discrimination (Task 3). This to our knowledge is the first demonstration of conditional discrimination in a cephalopod of the order Teuthida (i.e. squids). These six squid also showed significant learning abilities by demonstrating improvements in choosing the correct exit in subsequent trials within each task, and by completing Task 2 (testing with preference) and Task 3 (conditional discrimination) in significantly fewer trials compared to in Task 1 (testing against preference). The six squid that demonstrated conditional discrimination were represented by three individuals from control and three individuals from elevated CO_2_, indicating that elevated CO_2_ had no effect on the capacity of *S. lessoniana* to conditionally discriminate. Furthermore, elevated CO_2_ did not have an effect on any of the traits measured within this experiment including the number of trials taken to complete each task, time to exit the arena, and percentage of correct exit choices within each task. This suggests that elevated CO_2_ also has no effect on the learning or long-term memory of *S. lessoniana*. This experiment has brought to light some of the remarkable cognitive abilities of *S. lessoniana*, and suggests that seawater *p*CO_2_ projected for the end of the century under a worst case scenario does not inhibit these abilities.

Squid in this experiment demonstrated individual variation in their learning and conditional discrimination abilities, and this individual variation was greater than any potential effect of CO_2_ treatment. Importantly, individuals that passed all three tasks spent significantly more time in the arena during trials in Task 1 compared to individuals that failed to reach and pass Task 3. It is possible that spending more time within the arena gave the squid time to better assess their surroundings and commit visual cues to memory. Animals that failed to reach Task 3 were also more erratic in their Task 1 escapes in comparison to individuals that eventually demonstrated conditional discrimination. Throughout trials, squid that eventually demonstrated conditional discrimination typically slowly entered the arena and then made an exit choice after a brief pause somewhere between the two exits. In contrast, individuals that did not eventually demonstrate conditional discrimination typically moved more rapidly after exiting the habituation chamber, and jetted around the perimeter of the arena until reaching an exit. During Task 2, these rapidly jetting individuals had a mean percentage of correct exit choices of 51%, indicating complete randomness in their exit choice. Slower performing individuals that eventually demonstrated conditional discrimination chose the correct exit in Task 2 in a mean of 84% of their trials. This higher percentage of success, along with the significant increase in correct exit choice over successive trials, suggests that these individuals may have benefited from spending more time in the arena during Task 1 trials.

Individuals that were unable to demonstrate conditional discrimination completed Task 1 in approximately half as many trials compared to individuals that did demonstrate conditional discrimination by passing Task 3. It appears that performance during Task 1 was crucial for the squid to successfully reach and pass Task 3. A faster exit time and fewer number of trials for each task would seemingly indicate better performance. However, in this experiment it seems that it was important for individuals to spend sufficient time within the arena as well as to make a fair number of incorrect choices during Task 1 in order to understand the conditions of the arena. A drawback of this experiment is that once an individual passed Task 1, it was not presented that combination of visual cue and corresponding exit again until after it successfully passed Task 2 and reached the random trials. Animals that passed Task 1 in a very short number of trials (some in the minimum seven trials) may not have had sufficient trial and error experience in their learning process. Trial and error is an integral part of many learning processes, leading to sustained modifications of behaviour and improvements in complex tasks (Dayan & Balleine, 2002; Ruediger et al., 2012). Squid that completed Task 1 in a minimal number of trials may have benefited from a reintroduction to Task 1 if they were unable to complete Task 2 after a set number of trials.

Of the seven squid to reach Task 3, four were from the control treatment. However, one squid from control did not demonstrate conditional discrimination despite having reached Task 3. This squid, like individuals that failed Task 2, successfully chose the correct exit in only 50% of 26 total trials during Task 2. It seems likely that this individual was choosing the exit at random in Task 2 and met the passing criteria by chance. The lack of conditional discrimination among visual cues in this individual becomes apparent in the randomised trials of Task 3, in which only 35% of 60 trials did it choose the correct exit. Among animals that successfully demonstrated conditional discrimination, the mean percentage of correct exit choices was only slightly lower in Task 3 (80%) compared to in Task 2 (84%). A decrease in percentage of correct choices here was expected as the random aspect of Task 3 was a new concept for the squid. However, this difference was not as large as expected and shows advanced long-term memory, for up to 7 days, and context dependent discriminatory abilities.

The ability for cephalopods to conditionally discriminate and rapidly learn, especially during early life, is important in order to keep up with their fast-paced lifestyle (Dickel, Boal & Budelmann, 1999). Changes to these abilities could have adverse effects on squid predation, predator-avoidance, navigation/migration, and many other behaviours. This experiment provides no evidence that elevated CO_2_ has any effect on the learning abilities of *S. lessoniana*. However, the small sample size, large variation in individual performance, and high difficulty of the passing criteria (50% pass rate) in this study may have masked any subtle effects of elevated CO_2_ on learning. A power analysis revealed a probability of 0.22 of finding an effect of elevated CO_2_ if one were present. Perhaps potential differences in performance between CO_2_ treatments would be more detectible with a larger sample size, a more easily achievable associative learning test, and memory reinforcement in the first tasks. Nevertheless, the present study demonstrates advanced learning capabilities in squid, and no adverse effect of elevated CO_2_ exposure on learning.

Adverse effects on learning arising from elevated CO_2_ exposure has been demonstrated in damselfish and linked to inhibited GABA_A_ neurotransmitter function (Chivers et al., 2014). The effects of elevated CO_2_ on GABA_A_ also appear to be responsible for a wide range of altered behaviours, such as in anti-predator responses in molluscs (Watson et al., 2014) and anxiety, lateralisation, and olfactory preference in fishes (Nilsson et al., 2012; Hamilton, Holcombe & Tresguerres, 2014; Heuer & Grosell, 2014; Lai, Jutfelt & Nilsson, 2015; Schunter et al., 2018). Previously demonstrated effects of elevated CO_2_ on behaviour in *S. lessoniana* include increased activity and altered predatory behaviours (Spady, Munday & Watson, 2018), but the mechanism responsible for these changes has not been investigated. The importance of GABA_A_ receptors for learning in squid (Conti et al., 2013), and evidence of the lack of inhibited learning at elevated CO_2_ in the current experiment, suggests that previously demonstrated altered behaviours in *S. lessoniana* at elevated CO_2_ (Spady, Munday & Watson, 2018) may be due to a mechanism other than altered functioning of GABA_A_ receptors. It is important to investigate what mechanism is causing behavioural changes in order to understand why some important behaviours are altered, whereas other equally important behaviours are unaffected by elevated CO_2_.

In addition to learning, the relatively large size of cephalopod brains are needed for processing large amounts of sensory information (Young, 1988). Cephalopods, for example can have 20 million receptor cells in the eye, requiring significant processing power from the brain and giving individuals a large amount of visual information to manage (Hanlon & Messenger, 2018). The ability to process complex visual cues, such as during male to male agonistic behaviour, is of great importance to their health and survival, as well as to the rewards of reproductive opportunities. As previous studies on fish have shown elevated CO_2_ to inhibit learning via visual predator recognition (Ferrari et al., 2012), perhaps testing predator recognition learning at elevated CO_2_ would be a valuable approach for *S. lessoniana*. Also, as Ferrari et al. (2012) used pre-settlement juveniles to test learning, it could be worthwhile to investigate effects of elevated CO_2_ on learning in *S. lessoniana* as juveniles rather than adults. In this way, experiments could focus on naturally acquired learning during a life stage in which learning is more rapid and potentially more ecologically beneficial.

While the current study investigates learning in support of spatial orientation rather than in support of predator recognition, Jutfelt et al. (2013) tentatively reported inhibited learning in fish in an arena escape trial as a result of elevated CO_2_ exposure. While both studies measured arena escape behaviours related to spatial orientation, there are a number of differences in the design of the studies and what was measured. Jutfelt et al. (2013) found reduced arena exit times in control fish from the initial trial to the second trial 20 days later, whereas fish held at elevated CO_2_ showed no difference in exit times between the two trials. While this could be indicative of effects on learning, it could equally be a result of elevated CO_2_ effects on exploratory behaviours (as the experiment was intended to measure), boldness, or activity levels. The present study is strictly an investigation into the learning of *S. lessoniana* with repeated motivation-based trial and error training, and provides no evidence of inhibited learning from elevated CO_2_.

## Conclusions

The importance of context learning in animals is of considerable ecological significance (Balsam & Tomie, 1985), and in squid, leads to the development of many important behaviours such as escape response, prey capture, body patterning, spatial orientation, and social behaviours (Hanlon & Messenger, 2018). The results from this study indicate for the first time that squid are able to conditionally discriminate, further expanding the known range of taxa with this ability. Furthermore, we found no evidence that elevated CO_2_ levels projected for the end of this century and subsequent ocean acidification have any effects on learning behaviours of *S. lessoniana*. Future studies should investigate the effects of elevated CO_2_ on a wider range of learning behaviours and in a variety of cephalopod species and life stages to ensure that these cognitive abilities will remain unaffected under future ocean conditions.

## Supplemental Information

10.7717/peerj.9865/supp-1 Supplemental Information 1 Statistical outputs of variables and factors tested among all individuals and for only individuals that demonstrated conditional discrimination. Asterisks (*) next to p-values less than 0.05 indicate a relationship to be significant different.

## Additional Information and Declarations

### Field Study Permissions

The following information was supplied relating to field study approvals (i.e. approving body and any reference numbers): Collection permits from the Queensland Department of Agriculture, Fisheries and Forestry were obtained for animal collection (Permit Number: 170251).

### Data Availability

The following information was supplied regarding data availability: Data are available at JCU Research Database: Spady, B. (2020): Bigfin reef squid demonstrates capacity for conditional discrimination and projected future carbon dioxide has no effect on learning capabilities. James Cook University. (dataset). DOI 10.25903/5e968544ea4d6.
