# Supplementary material for: Bigfin reef squid demonstrate capacity for conditional discrimination and projected future carbon dioxide levels have no effect on learning capabilities
Source: PeerJ. 2020 Sep 29;8:e9865. doi: 10.7717/peerj.9865 (PMC7531335; doi:10.7717/peerj.9865)
Supplement: Supplemental Information 1 — Asterisks (*) next to p-values less than 0.05 indicate a relationship to be significant different. [file peerj-08-9865-s001.pdf]

Table S1 – Statistical outputs. Statistical outputs of variables and factors tested among all individuals and for only individuals that demonstrated conditional discrimination. Asterisks (*) next to p-values less than 0.05 indicate a relationship to be significant different.

Variable Tested	Factors	df	χ^2	p-value
**All individuals**				
Preference strength	Treatment	11	1.000	0.317
Ability to pass Task 2	Treatment	11	0.707	0.401
	Task 1 cue	11	2.464	0.116
	Preference strength	11	0.416	0.519
Ability to demonstrate conditional discrimination	Treatment	11	0.024	0.876
	Task 1 cue	11	5.967	0.015*
	Preference strength	11	0.279	0.597
Ability to pass Task (in individuals that reached Task 3)	Treatment	6	1.243	0.265
Ability to pass Task 2	Task 1 Time	11	6.705	0.010*
Ability to demonstrate conditional discrimination	Number of trials needed to pass Task 1	11	4.053	0.044*
**Individuals that demonstrated conditional discrimination**				
Number of trials needed to pass Task 1	Treatment	5	0.262	0.609
Number of trials needed to pass Task 2	Treatment	5	0.320	0.571
Number of trials needed to pass Task 3	Treatment	5	0.069	0.511
Time to exit arena in Task 1	Treatment	5	0.012	0.914
Time to exit arena in Task 2	Treatment	5	0.433	0.511
Time to exit arena in Task 3	Treatment	5	0.371	0.542
Improvements over successive trials in the time to exit arena during Task 1	Treatment	5	0.001	0.971
	Trial number	5	0.426	0.514
	Mantle length	5	0.073	0.788
	Preference strength	5	0.056	0.813

	Treatment * Trial number	5	0.909	0.340
Improvements over successive trials in correct exit choice during Task 1	Treatment	5	5.702	0.017*
	Trial number	5	5.807	0.016*
	Mantle length	5	5.551	0.018*
	Preference strength	5	6.969	0.008*
	Treatment * Trial number	5	0.073	0.787
Improvements over successive trials in the time to exit arena during Task 2	Treatment	5	3.249	0.071
	Trial number	5	0.431	0.511
	Mantle length	5	3.129	0.077
	Preference strength	5	0.591	0.442
	Treatment * Trial number	5	2.499	0.114
Improvements over successive trials in correct exit choice during Task 2	Treatment	5	0.001	0.981
	Trial number	5	6.617	0.010*
	Mantle length	5	0.663	0.416
	Preference strength	5	0.551	0.458
	Treatment * Trial number	5	1.960	0.162
Improvements over successive trials in the time to exit arena during Task 3	Treatment	5	0.045	0.831
	Trial number	5	1.499	0.221
	Mantle length	5	0.561	0.454
	Preference strength	5	0.491	0.484
	Treatment * Trial number	5	2.005	0.157
Improvements over successive trials in correct exit choice during Task 3	Treatment	5	0.143	0.705
	Trial number	5	3.117	0.078

	Mantle length	5	1.529	0.216
	Preference strength	5	0.284	0.594
	Treatment * Trial number	5	0.018	0.892
Improvements in number of trials needed to pass successive Tasks	Treatment	5	4.059	0.044*
	Task number	5	40.842	<0.001*
	Preference strength	5	8.677	0.003*
	Treatment * Task number	5	1.249	0.535
Improvements in time to exit arena in successive Tasks	Treatment	5	0.333	0.564
	Task number	5	4.446	0.108
	Preference strength	5	0.032	0.858
	Treatment * Task number	5	3.981	0.137
Improvements in percentage of trials with correct exit choice in successive Tasks	Treatment	5	0.331	0.565
	Task number	5	2.919	0.232
	Preference strength	5	0.144	0.705
	Treatment * Task number	5	2.103	0.350
